# Ovarian cancer beyond imaging: integration of AI and multiomics biomarkers

**DOI:** 10.1186/s41747-023-00364-7

**Published:** 2023-09-13

**Authors:** Sepideh Hatamikia, Stephanie Nougaret, Camilla Panico, Giacomo Avesani, Camilla Nero, Luca Boldrini, Evis Sala, Ramona Woitek

**Affiliations:** 1 Research Center for Medical Image Analysis and AI (MIAAI), Danube Private University, Krems, Austria; 2 Austrian Center for Medical Innovation and Technology (ACMIT), Wiener Neustadt, Austria; 3 Department of Radiology, Montpellier Cancer Institute, University of Montpellier, Montpellier, France; 4 Dipartimento di Diagnostica Per Immagini, Radioterapia Oncologica Ed Ematologia, Fondazione Policlinico Universitario A. Gemelli IRCCS, Rome, Italy; 5 Scienze Della Salute Della Donna, del bambino e Di Sanità Pubblica, Fondazione Policlinico Universitario A. Gemelli IRCCS, Rome, Italy; 6 Department of Radiology, University of Cambridge, Cambridge, UK; 7 Cancer Research UK Cambridge Centre, University of Cambridge, Cambridge, UK

**Keywords:** Artificial intelligence, Biomarkers, Diagnostic imaging, Ovarian neoplasms, Multiomics

## Abstract

High-grade serous ovarian cancer is the most lethal gynaecological malignancy. Detailed molecular studies have revealed marked intra-patient heterogeneity at the tumour microenvironment level, likely contributing to poor prognosis. Despite large quantities of clinical, molecular and imaging data on ovarian cancer being accumulated worldwide and the rise of high-throughput computing, data frequently remain siloed and are thus inaccessible for integrated analyses. Only a minority of studies on ovarian cancer have set out to harness artificial intelligence (AI) for the integration of multiomics data and for developing powerful algorithms that capture the characteristics of ovarian cancer at multiple scales and levels. Clinical data, serum markers, and imaging data were most frequently used, followed by genomics and transcriptomics. The current literature proves that integrative multiomics approaches outperform models based on single data types and indicates that imaging can be used for the longitudinal tracking of tumour heterogeneity in space and potentially over time. This review presents an overview of studies that integrated two or more data types to develop AI-based classifiers or prediction models.

**Relevance statement** Integrative multiomics models for ovarian cancer outperform models using single data types for classification, prognostication, and predictive tasks.

**Key points**

• This review presents studies using multiomics and artificial intelligence in ovarian cancer.

• Current literature proves that integrative multiomics outperform models using single data types.

• Around 60% of studies used a combination of imaging with clinical data.

• The combination of genomics and transcriptomics with imaging data was infrequently used.

**Graphical Abstract:**

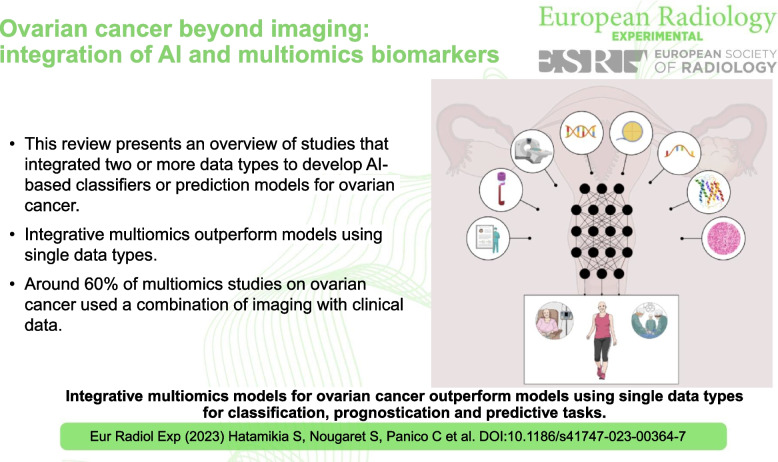

## Background

High-grade serous ovarian cancer (HGSOC) is the most common type of ovarian cancer and the most lethal gynaecologic malignancy, with 4.32 per 100,000 women predicted to die from ovarian cancer in the European Union in 2022 [1]. The disease typically presents at an advanced stage with ascites and extra ovarian spread (*i.e.,* peritoneal carcinomatosis) with multiple implants within the abdomen. Up-front treatment options comprise primary debulking surgery followed by platinum-based chemotherapy or neoadjuvant chemotherapy (NACT) with subsequent interval debulking surgery depending on disease extent and locations. Beyond chemotherapy, poly (ADP-ribose)polymerase and/or vascular endothelial growth factor inhibitors are used to treat ovarian cancer. Patient stratification and selection for the different clinical pathways are not entirely standardised across centres but also strongly depend on surgical expertise, training and facilities. Despite maximal surgical effort and molecular-driven maintenance therapy, many patients with ovarian cancer recur and eventually develop chemotherapy-resistant disease. Poor prognosis is underpinned by the loss of DNA repair mechanisms, resulting in high genomic intra-tumoural heterogeneity, early clonal evolution and rapid onset of chemoresistance [2, 3].

Currently, the evaluation of disease extent and response assessment in patients with HGSOC are based on the subjective analysis of cross-sectional imaging, *i.e.,* computed tomography (CT) and/or magnetic resonance imaging (MRI). CT of the abdomen, pelvis, and often also the chest is a key component in the multidisciplinary discussion leading up to a recommendation of either NACT and interval debulking surgery or primary debulking surgery. CT-based criteria suggesting low chances of optimal cytoreduction and therefore favouring NACT over primary debulking surgery are bulky or multifocal disease along the large or small bowel and the mesentery, bulky disease at the *porta hepatis*, along the liver surface including the gallbladder fossa, and nonresectable diaphragmatic or thoracic disease [4]. However, these criteria are subjective and not homogeneously applied across centres. Response evaluation criteria in solid tumors (RECIST) 1.1 is the mainstay of response assessment on CT scans of patients undergoing NACT [5]. Despite efforts to develop objective response criteria such as RECIST 1.1, interobserver variability, and subjective selection of target lesions remain major challenges that may affect response classifications and frequently require centralised image interpretation for drug registration trials [6]. There is an urgent need to obtain more robust imaging biomarkers for a better understanding of the initial presentation and successive monitoring of HGSOC during treatment that can be used to more effectively tailor therapy and ultimately improve outcomes. Radiomics has emerged as a tool for large-scale quantitative feature extraction from standard diagnostic imaging and holds great potential for developing image-derived biomarkers [7]. Recent advances in computational power have facilitated the use of deep learning (DL) and convolutional neural networks to automate image analysis further for tasks such as lesion classification, prognostication and response prediction.

To date, the genomic heterogeneity that characterises ovarian cancer, and HGSOC in particular, can only be captured spatially by sampling multiple instead of single disease sites and temporally by sampling the tumour at different time points during treatment which is hardly acceptable by patients. However, studies that harness artificial intelligence (AI) for integrating molecular omics with quantitative and standardised image analysis hold the potential for unravelling imaging signs of molecular heterogeneity that can then be used for the development of improved predictive and prognostic biomarkers. Cancer cells suffer deregulations at multiple levels including deoxyribonucleic acid (DNA), ribonucleic acid (RNA), proteins and metabolites; therefore, integrated multiomics data analysis is essential to fully understand the complexity of cancer and to capture features relevant to prognostication and prediction making. Although it has been shown that these approaches yield higher predictive performance when compared to studies focusing on single omics [8], only a minority of studies use imaging and radiomics integrated into a wider multiomics approach. With this review, we set out to provide an overview of research that has taken on the challenge of integrating at least two data types into AI-based prediction or classification models for ovarian cancer.

### Search strategy and eligibility criteria

Between October 2022 and January 2023, PubMed/MEDLINE, IEEE Xplore Digital Library, and Google Scholar were searched for studies that developed an AI algorithm for classification or prediction-making in ovarian cancer patients using multiple data types including radiological imaging. A controlled vocabulary supplemented with keywords such as radiomics, AI, imaging, multiomics, and data integration for ovarian cancer was used for the search. We limited the results to journal articles and conference proceedings. Conference abstracts, editorials, and letters to the editor were excluded from our search. Only English-language articles were considered.

### AI: machine learning and deep learning

AI is a field of computer science where computers mimic human intelligence and attempt to perform certain tasks that normally require human cognition, such as problem-solving and decision-making [9]. The two main fields of AI, machine learning (ML) and deep learning (DL), have shown higher performance than traditional approaches in the molecular characterisation of cancer, prognostication, diagnosis, patient classification, and prediction-making in various cancer types, including ovarian cancer [10, 11].

In the traditional paradigm of programming, AI tools use manually created programs that use given input data to produce the desired output. ML uses algorithms to automatically and iteratively learn from those data to perform a certain task, thus giving computers the ability to learn without being explicitly programmed. DL is a subset of ML, inspired by human artificial neural networks, which tries to mimic the learning process of the human brain. In ML, feature extraction (the process of transforming raw data set into relevant features) is handled manually, while the feature extraction process is fully automated in DL.

Of the 34 studies reviewed, 88% used ML, whereas only four studies used DL alone [8, 12–14] and two studies [15, 16] combined ML with DL techniques (Table 2). Three of the four studies that used only DL [8, 13, 14] combined genomics, epigenomics and transcriptomics. So far, only ML techniques have been proposed for predicting complete surgical cytoreduction and residual disease [17–19] while both ML and DL methods have been used for other general predictive and diagnostic goals/applications such as prediction of survival, recurrence, response to neoadjuvant chemotherapy, and differentiation between malignant and benign cancers and various cancer subtypes.

### AI and radiomics

Among the most frequently used ML algorithms for classification tasks in ovarian cancer are support vector machines, multilayer perceptron networks, decision tree, random forests, extreme gradient boosting (XGBoost), and logistic regression. These techniques make processing numerical features (such as clinical and demographic data or blood test results) relatively straightforward. However, medical images are multidimensional data and must be converted into numerical features to be used as input to such ML algorithms. Radiomics is a method for the computerised extraction of quantitative imaging features that allow the use of imaging data for machine learning; radiomics features are numerical descriptors of the shape, intensity, and texture of a structure such as a tumour on imaging [20]. Encoding the information captured on multidimensional images into radiomics feature vectors allows information from radiological data to be combined with other modalities (clinical data, genomics, etc.). Many radiomics features are highly correlated, therefore, feature selection methods are crucial as they improve the performance of the ML model, select the most relevant features and eliminate irrelevant and redundant features thus reducing the computational cost of modelling. Of all studies, 82% reviewed use feature selection as part of their models (Table 2).

One of the bottlenecks of many radiomics studies is the fact that radiomics feature extraction requires manual segmentation of the regions of interest, which is time-consuming and suffers from inter-observer variability. Differently, DL algorithms attempt to identify complex associations of original features using combinations of different deep neural layers and generate new features that can improve the performance of a particular classification task compared to the original features [14]. Convolutional neural networks are a common DL method and a type of network that is particularly appropriate for computer vision and image analysis for purposes such as image classification and automated feature extraction [21]. It is important to note that DL approaches require larger medical datasets compared to standard handcrafted radiomics and ML methods to efficiently fit the training model and produce the desired results.

### Integration of different data types

The most frequently used data in the reviewed ovarian cancer studies are clinical and demographic data. These data, even if not universally considered as proper “omics data”, are widely available, easy to collect, and not expensive and could be integrated into clinical care more easily than predictive models based on far more expensive DNA or RNA sequencing, DNA methylation, or proteomic data.

Interestingly, 50% of the studies reviewed here benefitted from high-throughput information from radiomic data, mainly obtained from CT (27%) followed by MRI (21%) and less frequently from ultrasound (US) (3%) (Table 1). All of these studies combined radiomic signatures with clinical data, and most of them also with serum biomarkers, whereas only very few took into account genomics [22–24] or histopathology [16, 25].

**Table 1:** Categorisation of the reviewed studies based on their used data type and target of the study

Reference, year	Clinical and demographics	Serum biomarkers (CA-125 etc.)	Imaging	Handcrafted radiomics	DL features	DNA	DNA methylation	Gene expression (RNA)	Pathology	Proteomics	Target
[17], 2022	Yes	Yes									Complete surgical cytoreduction
[26], 2020	Yes	Yes									Benign *versus* malignant
[18], 2015	Yes	Yes									Survival, complete surgical cytoreduction
[27], 2002	Yes	Yes									Benign *versus* malignant
[28], 2019	Yes	Yes									Clinical stage, histotype, residual tumour burden
[29], 2022	Yes	Yes									Postoperative critical care unit admission
[30], 2022	Yes	Yes									Benign *versus* malignant, determining pathological type, grade and clinical stage
[31], 2001	Yes	Yes	US								Benign *versus* malignant
[32], 2021	Yes	Yes	US	Yes							Benign *versus* borderline *versus* malignant
[33], 2021	Yes	Yes	PET/CT	Yes							Survival
[12], 2019	Yes	Yes	CT		Yes						Recurrence
[15], 2022	Yes		CT	Yes	Yes						Recurrence, BRCA mutation
[34], 2021	Yes		CT (semantic)								Survival
[35], 2021	Yes		CT	Yes							Recurrence
[36], 2022	Yes		CT	Yes							Survival
[16], 2022	Yes		CT	Yes	Yes				Yes		Response to neoadjuvant chemotherapy
[22], 2020	Yes		CT	Yes				Yes			Progression-free survival and platinum resistance (recurrence?)
[23], 2022			CT	Yes				Yes			Prediction of the hypoxia pattern
[24], 2021	Yes	Yes	CT	Yes		Yes					Platinum resistance (recurrence?)
[37], 2021	Yes	Yes	CT	Yes		ctDNA					Response to neoadjuvant chemotherapy
[25], 2022	Yes	Yes	MRI	Yes					Ki67		Recurrence
[38], 2021	Yes	Yes	MRI	Yes							Peritoneal metastases
[39], 2022	Yes	Yes	MRI	Yes							Recurrence
[40], 2021	Yes	Yes	MRI	Yes							Peritoneal metastases
[19], 2021	Yes	Yes	MRI	Yes							Residual disease, complete surgical cytoreduction
[41], 2022	Yes		MRI	Yes							Benign *versus* malignant
[42], 2022	Yes	Yes	MRI	Yes							Benign *versus* borderline
[43], 2018	Yes					Yes	Yes	Yes			Survival
[44], 2005	Yes							Yes			Response to neoadjuvant chemotherapy
[45], 2021	Yes					Yes	Yes	Yes			Response to neoadjuvant chemotherapy
[8] 2021					Yes	Yes	Yes	Yes			Renign *versus* malignant, survival
[13], 2021					Yes	Yes	Yes	Yes			Survival
[14], 2020					Yes	Yes	Yes	Yes			Subtyping
[46], 2021						Yes		Yes	Yes	Yes	Survival

*CA-125* Cancer antigen 125, *ctDNA* Circulating tumour DNA, *CT* Computed tomography, *DL* Deep learning, *DNA* Deoxyribonucleic acid, *MRI* Magnetic resonance imaging, *PET* Positron emission tomography, *RNA* Ribonucleic acid, *US* Ultrasound

**Fig. 1:**
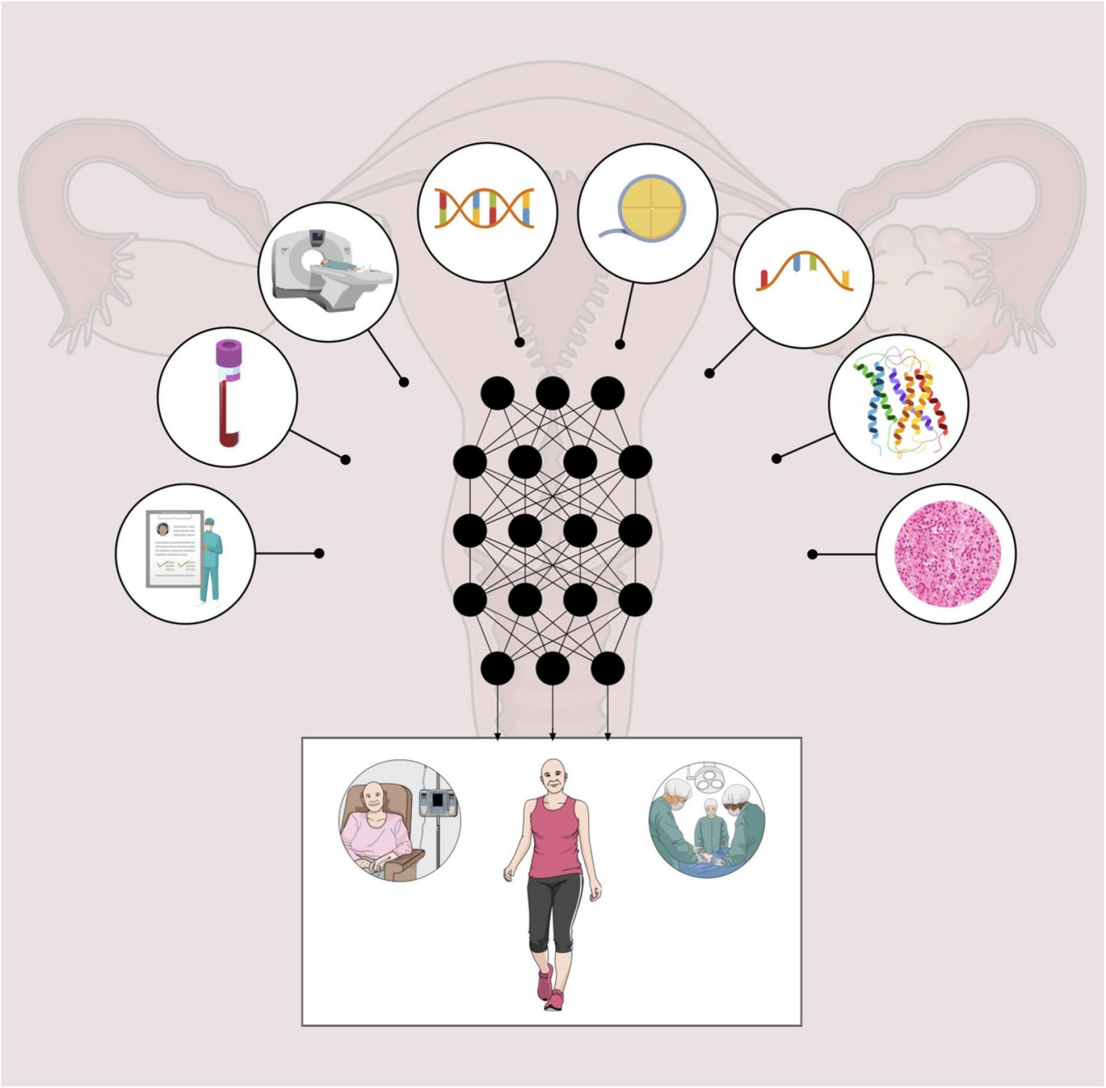
Multiomics studies in ovarian cancer to date use (circles from left to right) clinical data, serum biomarkers like cancer antigen 125 (CA-125), imaging including computed tomography (CT), magnetic resonance imaging (MRI), and ultrasound (US), genomics, epigenomics, transcriptomics, proteomics, and pathology data for the development of artificial intelligence-based cancer subtyping, lesion classifiers, and models for predicting patient outcome including response to chemotherapy, complete surgical cytoreduction, and survival (bottom panel)

In almost all studies reviewed, combining multiple data types improved the overall predictive performance compared with a single data type, with the only exception of Wang et al. [12], whose DL model based on CT data alone achieved higher prognostic performance in validation cohorts, in terms of accuracy and area under the curve (AUC) when compared to the combined model (clinical information and DL features). This result confirms the importance of integrating multiple data types to enrich the data space used by AI techniques (Fig. 1).

We found seven studies [17, 18, 26–31] that combined clinical characteristics and serum biomarkers for different research tasks (Tables 1 and 2). These studies used a variety of clinical features, including age, year of diagnosis and surgery, performance status, histological type, tumour grade and stage, timing of surgery, presence of ascites, site of bulky disease at surgery, size of the largest tumour deposit, tumour location, tumour diameter, outcome of surgery, and residual tumour size after initial surgery. In addition, various serum biomarkers such as cancer antigen 125 (CA-125), cancer antigen 153, alphafetoprotein, carcinoembryonic antigen, and carbohydrate antigen 19–9 were investigated. In these seven studies, different ML algorithms were used for various objectives, including differentiation between benign and malignant tumours [26, 27, 30], prediction of complete surgical cytoreduction, survival [17, 18], determination of clinical stage, histotype, residual tumour burden, histopathological cancer type [28, 30], and prediction of critical care unit admission [29]. In three reports [26, 27, 30], the logistic regression, multilayer perceptron, and XGBoost algorithms were able to achieve an accuracy of 0.97, 0.98, and 0.96, respectively. In two reports [27, 30], no feature selection method was used. Two studies [17, 18] reported an accuracy of up to 0.87 and 0.73, respectively, for predicting complete surgical cytoreduction using XGBoost and artificial neural network algorithms. In two studies [28, 30], higher accuracy was obtained in distinguishing benign from malignant lesions (0.97 and 0.96, respectively) compared with clinical stage determination (0.76 and 0.68, respectively). Except for one study [27], all studies used data sets of more than 290 patients.

**Table 2:** Summary of the data set sizes, models, features selection methods and performance metrics for the same publications as shown in Table 1 (combining at least two data types for model development)

Reference	Patient data Total, training, test	AI approach	Model used	Feature selection method	Validation method	Performance
[17]	571, 399, 172	ML	eXtreme Gradient Boosting (XGBoost)	Shapley Additive explanations (SHAP) values	Training and test cohorts (70:30% ratio), fivefold stratified cross-validation (CV)	Area under the curve (AUC) on: Test: 0.866
[26]	349, 235, 114	ML	Decision tree (DT), Logistic regression (LR), Risk of ovarian malignancy algorithm (ROMA)	Minimum Redundancy–Maximum Relevance (MRMR), Relief	tenfold CV	The highest AUC using DT and LR on: Training: 0.88, 0.87 Test: 0.949, 0.969
[18]	668	ML	DT, artificial neural network (ANN) and Bayesian network (BN), support vector machines (SVM), Naive Bayes (NB), K-nearest neighbor (KNN), and LR	−	tenfold CV	The highest accuracy (ACC) and AUC using ANN on: Test (prediction of survival) = 93% and 0.74 Test (complete surgical cytoreduction) = 77% and 0.73
[27]	55	ML	Multi-layer perceptron (MLP) networks	−	Not mentioned	ACC on: Test = 92.9% Area under the curve on: Test: 0.98
[28]	435, 334, 101	ML	Gradient boosting machine (GBM), SVM, Random forest (RF), Conditional RF (CRF), NB, ANN, and Elastic net (EN)	Gini index	tenfold CV	The highest ACC and AUC using RF on: Test (benign *versus* malignant) = 92.4% and 0.968 Test (clinical stage) = 69% and 0.76
[29]	291, 175, 116	ML	K-nearest neighbors (KNN), ANN, linear discriminant analysis (LDA), and quadratic discriminant analysis (QDA), LR	Forward selection and backward stepwise regression and correlation analysis	Random split into training and test cohorts (60:40% ratio), Leave-one-out (LOO) CV	The highest ACC, sensitivity, specificity, F-score by LDA and QDA on: Training: 0.97, 0.96, 0.97, 0.96 and 0.97, 1.00, 0.97, 0.98 Test: 0.90, 0.93, 0.89, 0.91 and 0.93, 0.93, 0.93, 0.93
[30]	532, 372, 160	ML	LR, DT, RF, Adaptive boosting (AdaBoost), XGBoost, GBM, NB, SVM, EN, and ANN	−	fivefold CV	The highest AUC using XGBoost and test data for: Distinguishing cancer and benign: 0.958 Determining pathological type: 0.792 Determining grade: 0.819 Determining clinical stage: 0.68
[31]	425, 265, 160	ML	MLP, generalised regression networks (GRNNs), LR	Stepwise multivariate LR analysis	Stratified sevenfold CV	The highest AUC using MLP, GRNN, LR on training: 0.975, 0.966, 0.966 Test: 0.924, 0.911, 0.908
[32]	265, 185, 80	ML	LR-based nomogram	Least absolute shrinkage and selection operator (LASSO)	Split into training and test cohorts (70:30% ratio), 10-Fold CV	The AUC for ultrasound (US)-based radiomics models for discrimination benign *versus* borderline and malignant (Task 1) and discrimination between borderline *versus* malignant (Task2): Test: 0.877 and 0.839 The AUC of combined model based clinical features and radiomics for Task 1 and Task 2: Test: 0.914 and 0.890
[33]	261, 182, 79	ML	Multivariate Cox regression and nomogram	LASSO	fivefold CV	The beast performance was using integration of clinical and PET radiomics features: Training: C-index = 0.70, 95% CI 0.68–0.72 Validation: C-index = 0.70, 95% CI 0.66–0.74
[12]	245, 151, 94	DL	Model incorporating the DL feature and Cox proportional hazard (Cox-PH) regression and a clinical model	-	Training and validation cohorts	The AUC of the DL model on: Two validation cohorts: 0.772 and 0.825
[15]	218, different train and test data division	ML and DL	Convolutional Neural Network (CNN), Penalised LR, RF, SVM	ComBat harmonisation and the reproducibility analysis performed with the ANOVA	fivefold CV	The best AUC using radiomics ML model: Test (prediction of BRCA mutation): between 0.46 and 0.59 Test (prediction of 1-year relapse): between 0.46 and 0.56 The AUC using DL model: Test (prediction of BRCA mutation): 0.48 Test (prediction of 1-year relapse): 0.50 Integration of clinical variables with radiomics model improved the performance of the BRCA mutation prediction (AUC: 0.74)
[34]	209, 167, 42	ML	SVM, KNN, Ensemble Classifiers, NB, LR	Exhaustive search, the-chi square test of independence and the MRMR method	Training and test cohorts split (80: 20% ratio) with repeated random sampling, tenfold CV	The AUC of achieving cancer-free state Test: AUC 0.63 The AUC of Mid-term overall survival Test: AUC between 0.63 and 0.66 The model recall and precision greater than 80% were achieved
[35]	256, 179, 77	ML	SVM, nomogram	Recursive feature elimination	Training and test cohorts (70:30% ratio)	The AUC for radiomics model, clinical model, and combined model: Training: 0.715, 0.632, 0.749 Test: 0.717, 0.691, 0.769 The radiomics model achieved AUCs of 0.715
[36]	119, 80, 39	ML	Nomogram based on multivariable Cox regression analysis	LASSO	tenfold CV	The C-indexes of radiomics model and radiomic clinical nomogram on: Training: 0.694, 0.754 Validation: 0.709, 0.727
[16]	444, 404, 40	ML, DL	Cox Proportional-Hazards Model, ResNet-18 convolutional neural network	Multivariable significance on features using Cox models	fourfold slide-wise CV	The concordance indices using radiomics on: Training: 0.55 (95% CI 0.549–0.554) Test: 0.53 (95% CI 0.517–0.547) Using histology data: Training: of 0.56 (95% CI 0.559–0.564) Test: 0.54 (95% CI 0.527–0.560) Using combination of both imaging and histopathological data: Test: 0.62 (95% CI 0.604–0.638) Using genomic, radiomic and histopathological data: Test: 0.61 (95% CI 0.594–0.625)
[22]	75	ML	SVM	Recursive feature elimination	threefold CV	The Concordance probability estimates (CPE) of the radiomics-clinical-genomic on: Test (progression-free survival): 0.695 (95% CI of 0.64 to 0.75) Test (platinum resistance classification): 0.78 (95% CI 0.77 to 0.80)
[23]	59, 40, 19	ML	Multivariate Cox regression analysis	LASSO and correlation coefficients	Training and test cohorts (~ 70:30% ratio)	The AUC of the radiogenomic model on: training: 0.900 Test: 0.703
[24]	102, 71, 31	ML	RF, SVM	LASSO	Random separation of data into training and test cohorts (70:30% ratio)	The AUC of the radiomics model, genomics model and the radiomics-clinicopathological-genomic model: Training: 0.874, 0.843, 0.993 Validation: 0.832, 0.815, 0.967
[37]	134, 72, 62	ML	EN, Support vector regression, RF	Collinearity reduction and univariable selection, feature’s coefficients and impurity-based feature importance	fivefold CV	Radiomics integration model produced Spearman *r* = 0.32, *p* = 0.04 and with a reduction in mean square error MSE of 8%
[25]	186, 130, 56	ML	SVM, LR, RF, nomogram constructed based on multivariate logistic regression analysis	Pearson correlation coefficient (PCC), recursive feature elimination (RFE), Kruskal–Wallis (KW) test and Relief	The dataset was split based on unsupervised K-means clustering algorithm into training and test (70:30% ratio), fivefold CV	The highest AUC using radiomics-clinical nomogram on: Training: 0.866 Validation: 0.818
[38]	89, 54, 35	ML	Nomogram constructed based on the multivariate logistic regression	LASSO and a backward stepwise multivariate LR, considering Akaike’s information criterion (AIC) as the stopping rule	threefold cross CV	The AUC using radiomics model and radiomic-pelvic fluid-CA-125 model on: Training: 0.963, 0.969 Validation: 0.928, 0.944
[39]	141, 99, 42	ML	Multivariate LR	Features with Intraclass correlation coefficients (ICCs) > 0.75 were retained, LASSO	Random separation of data into training and test cohorts (70:30% ratio)	The AUC using clinical model, multi-radiomics model and combined model on: Training: 0.76, 0.78, 0.83 Validation: 0.67, 0.74, 0.78
[40]	86	ML	Nomogram based on multi-factor LR	MRMR, LASSO	tenfold CV	The AUC of the radiomics model, clinical model and radiomics nomogram based on combining radiomics characteristics and clinicopathological risk factors on: Test: 0.846, 0.858, 0.902
[19]	217, 160, 57	ML	Nomogram based on multivariable LR	MRMR, LASSO	fourfold CV	The AUC using the clinico-radiological, radiomic and combined model on: Test: 0.623, 0.744, 0.803
[41]	148, 118, 30	ML	XGBoost	LASSO	Stratified random separation of data into training and test cohorts (80:20% ratio) and tenfold CV	The AUC, accuracy, precision, sensitivity of clinical, radiomic and combined models on: Test: 0.847, 0.774, 0.769, 0.714, and 0.807, 0.677, 0.643, 0.643, and 0.954, 0.839, 0.909, and 0.714, respectively
[42]	417, 387, 30	ML	LR, SVM, RF, NB	LASSO, Spearman correlation, Univariate analysis	Stratified random separation of data into training and test cohorts (80:20% ratio) and tenfold CV	The AUC of the radiomics, clinic-radiological and combined model using LR on: Test: 0.82, 0.79, 0.86
[43]	215, 172, 43	ML	RF, XGBoost, LR	A modification of MRMR	fivefold CV	The AUC using methylation data alone model, CAN model, RNA-Seq model and integrated multi-omics data model on: Test: 0.53, 0.64, 0.66, 0.70
[44]	135	ML	SVM	*t*-test statistical analysis	LOO CV	The average accuracy of integrated model (clinical information and gene expression data) increases by 19.56% and 45.08% compared to model using only gene expression data on two different datasets, respectively
[45]	88	ML	Multivariate regression	Univariate ANOVA, LASSO	tenfold CV	By integrating clinical and genomic variables an AUC over 95% was achieved
[8]	Datasets with three different sample sizes (292, 459, and 481)	DL	Maximum Mean Discrepancy Variational Autoencoder (MMD-VAE)	−	Random separation of data into training and test cohorts (60:40% ratio) and fivefold CV	The AUC using CNV, mRNA, Methylation, CNV_mRNA, mRNA_methylation, and CNV_mRNA_methylation on: Test: 54.3, 93.8, 75.2, 93.7, 93.2, 95.5
[13]	1314	DL	Deep neural networks with novel divergence-based consensus regularisation	Principal component analysis (PCA), unsupervised univariate feature selection by variance	Stratified fourfold CV with 60% training, 15% validation, and 25% testing data in each fold	Using PCA and multi-omics a higher performance (C-Index of 0.571 ± 0.036) was achieved compared to models using single omics
[14]	483, 298, 185	DL	A novel deep learning-based framework based on denoising Autoencoder, K-means and L1-penalised logistic regression	−	−	34 biomarkers and 19 (Kyoto Encyclopedia of Genes and Genomes) (KEGG) pathways associated with ovarian cancer were identified
[46]	321, 115, 206	ML	RF, LR, NB, DT, SVM, KNN, Gradient boosting decision tree (GBDT), Adaptive boosting (AdaBoost)	Gradient boosting (GB), LASSO	fivefold CV	AUC using histopathological image features, a model combining image features and genomics and multi-omics model including all features on: Validation: 0.703, 0.834, 0.911

*ACC* Accuracy, *AdaBoost* Adaptive boosting, *AIC* Akaike’s information criterion, *ANN* Artificial neural network, *AUC* Area under the curve, *BN* Bayesian network, *BRCA* Breast cancer, *CNV* Copy number variation, *Cox-PH* Cox proportional hazard, *CNN* Convolutional neural network, *CRF* Conditional random forest, *CV* Cross-validation, *DT* Decision tree, *EN* Elastic net, *GB* Gradient boosting, *GBDT* Gradient boosting decision tree, *GRNNs* Generalised regression networks, *GBM* Gradient boosting machine, *ICC* Intraclass correlation coefficients, *KEGG* Kyoto Encyclopedia of Genes and Genomes, *KNN* K-nearest neighbour, *KW* Kruskal–Wallis, *LASSO* Least absolute shrinkage and selection operator, *LDA* Linear discriminant analysis, *LOO* Leave-one-out, *LR* Logistic regression, *ML* Machine learning, *MLP* Multi-layer perceptron, *MMD-VAE* Maximum mean discrepancy variational autoencoder, *MRMR* Minimum redundancy–maximum relevance, *mRNA* Messenger ribonucleic acid, *NB* Naive Bayes, *PCA* Principal component analysis, *PCC* Pearson correlation coefficient, *PET* Positron emission tomography, *RF* Random forest, *RFE* Recursive feature elimination, *ROMA* Risk of ovarian malignancy algorithm, *QDA* Quadratic discriminant analysis, *SHAP* Shapley additive explanations, *SVM* Support vector machines, *US* Ultrasound, *XGBoost* eXtreme gradient boosting

### Imaging and other omics

To date, twenty studies have combined imaging data with other data types [12, 15, 16, 19, 22–25, 31–42] (Tables 1 and 2). Fifteen of these studies combined imaging data with clinical features and serum biomarkers [12, 15, 19, 31–36, 38–42]; two studies combined imaging data with clinical data and transcriptomics (gene expression; RNA) [22, 23]; two studies combined imaging data with clinical data, serum biomarkers, and histopathology data [16, 25]; and two studies combined imaging data with clinical data, serum biomarkers, and genomics (DNA and circulating tumour DNA) [24, 37]. CT [12, 15, 16, 22–24, 34–36] and MRI [19, 25, 38–42] were the most commonly used imaging modalities, while US and positron emission tomography (PET)/CT were used in only three studies [31–33]. Radiomics was used in all studies with imaging data, except for three studies that used either colour Doppler US and morphologic descriptors [31] and semantic CT features [34] or extracted DL features from images [12].

All studies used ML algorithms, except for one that used only DL algorithms [12]. Two studies used both ML and DL models on image data [15, 16].

The targets of the reviewed studies combining imaging data and other omics were distinguishing between benign, malignant, and borderline tumours [31, 32, 41, 42]; prediction of survival [22, 33, 34, 36]; prediction of recurrence and platinum resistance [12, 15, 22, 24, 25, 35, 39]; prediction of response to neoadjuvant chemotherapy [16, 37]; prediction of hypoxia [23]; prediction of peritoneal metastases [38, 40]; and prediction of complete surgical cytoreduction [19]. Integration of clinical variables with radiomics improved the performance of BRCA mutation prediction and achieved an AUC of 0.74 compared to only 0.62 on training data and 0.59 on test data for models based on radiomics alone [15]. The highest AUC (0.81) was also reported using a radiomics-clinical nomogram in [25] compared with radiomics or a clinical model alone. In [32], the AUC for US-based radiomics model combined with clinical features reached 0.91, compared to 0.88 for the radiomics model alone. The best performance (C-index 0.70, 95% confidence interval 0.66−0.74) was achieved by integrating clinical and PET radiomics features in [33] compared to using radiomics or clinical features alone. A higher AUC metric was also reported in [36] using a combined model (0.77) compared to using only radiomics (0.72) or clinical data (0.69). In [38], authors reported a higher performance using a radiomic-pelvic fluid-CA-125 model (AUC 0.94) compared with a model using radiomics alone (AUC 0.92). A higher AUC was also achieved using a combined model (0.78) compared to the clinical model (0.67) and multiradiomics model (0.74) [39]. A study [40] combined model based on radiomics and clinicopathological risk factors lead to an absolute increase of 5% in AUC compared with clinical data or radiomics only. A combined model based on radiomics and clinical features achieved an absolute increase in AUC of 10% and 15%, respectively, when compared to a clinical and radiomics model alone [41]. In addition, combining radiomics signatures with other types of data has also shown promise, *e.g.,* a radiomics-histopathological model [16], a radiomics-clinical-genomic model [22], a radiogenomic model [23], a radiomics-clinicopathological-genomic model [24], and a radiomics-clinical-radiological characteristics model [42] showed higher performance compared to using radiomics, clinical, genomic, and histological models alone (details of model performance can be found in Table 2). However, there is still scope for optimising the different building blocks of radiomics pipelines to improve the AUC also of integrative multiomics predictors.

### Genomics, epigenomics, transcriptomics, and other omics

We found seven more studies that integrated genomics and epigenomics (DNA and DNA methylation), transcriptomics (gene expression, RNA, and other omics data [8, 13, 14, 43–46] (Tables 1 and 2). Three studies combined genomics, epigenomics and transcriptomics for benign *versus* malignant differentiation [8], survival prediction [13], and subtyping [14]. Two studies combined genomics, epigenomics, and transcriptomics with clinical data to predict survival [43] and to predict response to neoadjuvant chemotherapy [45]. In addition, one study combined transcriptomics with clinical data to predict response to neoadjuvant chemotherapy [44]. In another study [46], the authors combined genomics, transcriptomics, pathology, and proteomics data to predict survival in ovarian cancer.

Of these seven studies, four [43–46] used ML and three [8, 13, 14] used DL. In [8], the authors reported that a combination of copy number variation, mRNA, and methylation data (AUC 0.96) outperformed using copy number variation (AUC 0.54), mRNA (AUC 0.94), and methylation (AUC 0.75) data alone. A multiomics model also achieved a higher performance (C-Index 0.571 ± 0.036, mean ± standard deviation) compared to models using single omics in [13]. In [44], an integrated model based on clinical information and gene expression data increased the accuracy by more than 19% and 45% compared to a model using only gene expression data on two different datasets. An AUC over 0.95 was achieved by integrating clinical and genomic variables [45]. Some authors [46] achieved the best accuracy using a multiomics model combining histopathological image features and genomics (AUC 0.91) compared with the model using only histopathological image features (AUC 0.70). In addition, an integrated multiomics model based on DNA methylation, copy number alteration, and RNA achieved a higher AUC (0.70) than when using methylation data (AUC 0.53), copy number alteration (AUC 0.64), and RNA data alone (AUC 0.66).

### AI and multiomics-based heterogeneity analysis

Genomic studies of multiple samples from single patients have allowed detailed intra-patient inter-site heterogeneity studies and revealed the diverse patterns of clonal spread of HGSOC which are thought to shape the local tumour immune-microenvironment, to affect sensitivity to treatment and, therefore, to be prognostically relevant [2, 47, 48]. CT of the abdomen and pelvis is central to the clinical pathway in patients with HGSOC and provides a snapshot of the multisite disease burden in patients with advanced disease.

Radiomics allows to non-invasively quantify this inter-site heterogeneity and the integration of radiomics with molecular omics provides a unique opportunity for decoding the link between heterogeneity on imaging and at the molecular and cellular level [49]. Vargas et al. [50] developed CT-radiomics-based spatial heterogeneity metrics across multiple metastatic lesions and integrated these imaging-based heterogeneity metrics with clinical variables and genomics to predict survival and platinum resistance [22]. Of note, this integrated multiomics predictor outperformed other models based on fewer data types and also a multiomic model that included radiomics but did not take into account inter-site intra-patient heterogeneity. Besides radiomics, also multiparametric MRI and ^18^F-fluoro-deoxy-glucose-PET have been shown to hold great potential for an improved understanding of inter-site heterogeneity as clusters based on imaging-derived diffusivity, vascularity and metabolic parameters were associated with patterns of hypoxia on immunihistochemistry and distinct genetic alterations [51].

## Discussion

Over the past two decades, fast and affordable sequencing has revolutionised genomic, epigenomic, and transcriptomic research, introducing unprecedented innovations in all the disciplines of cancer care ranging from gynaecological oncology to radiation therapy. Improvements in computational power have similarly changed the landscape of imaging research with exponentially increasing publications on radiomics and AI [7, 52]. The literature reviewed here demonstrates the vast potential multiomics data integration holds for improving patient care and outcome. Integration of radiomics and clinical information consistently outperformed models using radiomics or clinical models alone [15, 19, 25, 32, 33, 35, 36, 38–41]. In addition, the integration of radiomics with other types of data such as histopathological, genomic, and clinicopathological data [16, 22–24] improved performance, illustrating the added value of combining radiomics features in the developed models. Furthermore, in studies that integrated genomics and epigenomics, transcriptomics, and other omics data [8, 13, 14, 43–46], integration of multiomics data improved the results compared to single omics and an AUC of up to 0.95 was achieved for the test data sets [45].

Although genomic features such as homologous recombination deficiency have shown significant therapeutic implications in ovarian cancer, their assessment is not yet integrated into clinical practice and can be challenging and expensive and is still not refundable in many countries. To date, no imaging-based classifiers or predictors of outcome are currently being used routinely in clinics. Their implementation could be facilitated with easily usable, economically sustainable and effective methods. They hold the promise of overcoming many of the above-mentioned issues but limitations in the design and execution of some of the literature on radiomics and AI are at least partially contributing to the current situation. The lack of independent external datasets to evaluate AI models in a large part of the studies reviewed here is representative of a shortcoming of many radiomics studies. The use of independent data sets ideally from different institutions with different patient demographics, and socioeconomics and with imaging studies acquired using different scanners and vendors is highly recommended to overcome this limitation. Secure sharing of pseudonymised or anonymised data sets as well as AI models between research institutions, for example in a federated setup, is one way of ensuring the publication and distribution of highly generalisable models and could increase the chances of timely integration into clinical workflows, thanks to the robust replicability of these experiments.

The majority of studies also lacks detailed descriptions of the software and source code to enable the independent reproduction of results, an issue also encountered in other areas of oncological imaging and AI [53]. In addition, a variety of different metrics are used in AI research to evaluate and indicate diagnostic and predictive power, for example, metrics such as accuracy, AUC, sensitivity, specificity, F-score, C-index, recall, and dice similarity coefficient, limiting the comparative interpretability of the existing studies (Table 2). The use of common and standardised metrics would facilitate quantitative comparisons of models across different cohorts and institutions, significantly increasing the clinical impact of these decision-support tools.

The use of sufficient and common metrics should also be considered in future AI research. The overwhelming majority of studies reviewed here showed a benefit of multiomics data integration over limiting classification or prediction model building to one data type only. Therefore, the future work in this field should focus on data integration.

The association of genomic intrapatient heterogeneity in ovarian cancer and prognosis has been established and imaging is a well-suited tool for non-invasively assessing heterogeneity between tumour sites and tracking it over time. However, the integration of heterogeneity studies including multiomics is only in its infancy but has already shown advantages over less comprehensive analyses in terms of predictive power [22]. Besides the well-established whole genome sequencing of tumour DNA, more innovative approaches like the assessment of circulating tumour DNA and proteomics are more rarely encountered but merit attention [37, 54]. CA-125 has a well-established role in the diagnosis and management of ovarian cancer but lacks both sensitivity and specificity and circulating tumour DNA holds the potential to overcome this limitation [55]. Radiomics-based habitats have been used for targeting tissue sampling under ultrasound guidance, a technique that holds the potential to allow the integration of molecular tissue studies and radiomics with the advantage of limiting the exposure of the patient to ionising radiation and improving the design of clinical trials and their end-points [56, 57].

## Conclusions

AI tools for integrating multiomics data for tasks such as adnexal lesion classification and outcome prediction in ovarian cancer were reviewed in this review. The current literature proves that AI-based tools based on multiomics data integration are more than the sum of their parts and clearly outperform single-omic data sets. Clinical data, serum markers and imaging data (predominantly using handcrafted radiomics) were the data most frequently paired up, followed by genomics and transcriptomics.

The latter two were only infrequently combined with imaging data, highlighting a current gap in the available literature. Only rarely the AI methods have been described in enough detail that would allow the reproduction of the results. Also, sharing data and analysis algorithms is uncommon thus hampering independent validation of results, a prerequisite for AI tools to be considered for clinical use. Heterogeneity at the genomic and tumour-microenvironment level represents a hallmark of ovarian cancer, likely contributing to its poor prognosis. Pivotal studies have shown that imaging holds the key to describe and understand heterogeneity, not only at the spatial but also temporal level with multiple scans being routinely performed throughout our patients’ path of care, allowing the setup of innovative therapeutic solutions and potentially improving treatment outcomes.

## Data Availability

Not applicable (article is a narrative review).

## Abbreviations

AI: Artificial intelligence
AUC: Area under the curve
CA-125: Cancer antigen 125
CT: Computed tomography
DL: Deep learning
DNA: Deoxyribonucleic acid
HGSOC: High-grade serous ovarian cancer
ML: Machine learning
MRI: Magnetic resonance imaging
NACT: Neoadjuvant chemotherapy
PET: Positron emission tomography
RECIST: Response evaluation criteria in solid tumors
RNA: Ribonucleic acid
US: Ultrasound
XGBoost: Extreme gradient boosting
